# A Transcriptional Profile of Aging in the Human Kidney

**DOI:** 10.1371/journal.pbio.0020427

**Published:** 2004-11-30

**Authors:** Graham E. J Rodwell, Rebecca Sonu, Jacob M Zahn, James Lund, Julie Wilhelmy, Lingli Wang, Wenzhong Xiao, Michael Mindrinos, Emily Crane, Eran Segal, Bryan D Myers, James D Brooks, Ronald W Davis, John Higgins, Art B Owen, Stuart K Kim

**Affiliations:** **1**Division of Nephrology, Stanford University Medical CenterStanford, CaliforniaUnited States of America; **2**Department of Developmental Biology, Stanford University Medical CenterStanford, CaliforniaUnited States of America; **3**Department of Biochemistry, Stanford University Medical CenterStanford, CaliforniaUnited States of America; **4**Department of Pathology, Stanford University Medical CenterStanford, CaliforniaUnited States of America; **5**Department of Computer Science, Stanford University Medical CenterStanford, CaliforniaUnited States of America; **6**Department of Urology, Stanford University Medical CenterStanford, CaliforniaUnited States of America; **7**Department of Genetics, Stanford University Medical CenterStanford, CaliforniaUnited States of America; **8**Department of Statistics, Stanford University Medical CenterStanford, CaliforniaUnited States of America

## Abstract

In this study, we found 985 genes that change expression in the cortex and the medulla of the kidney with age. Some of the genes whose transcripts increase in abundance with age are known to be specifically expressed in immune cells, suggesting that immune surveillance or inflammation increases with age. The age-regulated genes show a similar aging profile in the cortex and the medulla, suggesting a common underlying mechanism for aging. Expression profiles of these age-regulated genes mark not only age, but also the relative health and physiology of the kidney in older individuals. Finally, the set of aging-regulated kidney genes suggests specific mechanisms and pathways that may play a role in kidney degeneration with age.

## Abstract 2

A study of human aging in the kidney reveals similar changes in the transcriptional profile in cortex and medulla, suggesting that a common underlying aging process is taking place

## Introduction

Aging affects nearly all organisms and is a major risk factor in most human diseases. Recent work has begun to uncover molecular mechanisms that specify lifespan and to identify alterations in cellular physiology that occur at the end of life (Tissenbaum and Guarente 2002). For example, oxidative damage caused by the generation of free radicals in the mitochondria has been found to hasten aging by causing an accumulation of damaged cellular components (Droge 2003). Telomere shortening may also play a role in aging by preventing DNA replication and cell division in later years (Hasty et al. 2003). Genetic studies have identified many genes that play a role in specifying lifespan. For example, mutations in yeast *sir2* (chromatin regulator), worm *daf-2* (insulin-like growth factor receptor), fly *methuselah* (tyrosine kinase receptor), mouse *p53,* and the human Werner's syndrome gene (DNA helicase) cause dramatic changes in lifespan (Guarente and Kenyon 2000). Several aging mechanisms alter longevity in multiple organisms. For example, mutations in the gene encoding insulin-like growth factor receptor alter lifespan in worms, flies, and mice, indicating that an endocrine signaling pathway has a conserved role in aging (Hekimi and Guarente 2003).

Genetic studies have shown that aging can be slowed in mutants that are defective in a wide range of cellular processes (such as mitochondrial function, chromatin regulation, insulin signaling, transcriptional regulation, and genome stability). This indicates that aging is a complex process driven by diverse molecular pathways and biochemical events. As such, a powerful approach to study aging is to use systems biology, which allows a multitude of factors affecting aging to be analyzed in parallel. For example, DNA microarrays and gene expression chips have been used to perform a genome-wide analysis of changes in gene expression in old age. Extensive studies in Caenorhabditis elegans and Drosophila melanogaster have identified hundreds of age-regulated genes (Hill et al. 2000; Zou et al. 2000; Lund et al. 2002; Pletcher et al. 2002; Murphy et al. 2003). Several studies have described age-regulated genes in the muscle and brain of mice (Lee et al. 1999, 2000) and the retina and muscle of humans (Yoshida et al. 2002; Welle et al. 2003, 2004). These age-regulated genes may serve as markers of aging, enabling one to assess physiological age independently of chronological age. Analysis of the functions of these age-regulated genes has identified specific biochemical mechanisms that change toward the end of life.

A key question still unresolved is to what extent the mechanisms of aging are conserved between species with vastly different lifespans. Some studies suggest that similar mechanisms are involved in aging in many species. For example, caloric restriction extends lifespan in yeast, worms, flies, mice, and primates (Weindruch 2003). Additionally, signaling through the insulin-like growth factor pathway, chromatin regulation by *sir2,* and oxidative damage have each been shown to affect lifespan in diverse model organisms (Tissenbaum and Guarente 2002). Other studies emphasize that changes occurring at the end of life are unlikely to be evolutionarily conserved (Kirkwood and Austad 2000). In the wild, very few animals (including humans) survive to their maximal biological lifespan. Thus, the changes in physiology that occur in very old animals have minimal effects on the fitness of individuals, and are unlikely to be evolutionarily conserved. Therefore, aging is likely to be species-specific, and studies of old age in model organisms are unlikely to be relevant to humans.

We have begun our studies of human aging by focusing on the kidney, an organ that shows a quantifiable decline in function with age. One of the primary functions of the kidney is to remove toxins from the blood, which involves filtering plasma through specialized capillary beds (glomeruli) in the renal cortex. The primary function of the tubules within the medulla is to concentrate or dilute the urine so as to maintain fluid balance. The major age-related change in kidney function is a 25% decline in the glomerular filtration rate starting at age 40 (Hoang et al. 2003). The ability of the medulla to concentrate urine declines progressively with age.

In this study, we present a molecular portrait of the aging process in the human kidney by analyzing gene expression as a function of age on a genome-wide scale. We show that age regulation is similar in the cortex and the medulla, and that age-regulated genes in the kidney are broadly expressed. We show that the expression profiles of age-regulated genes correlate well with the morphological and physiological state of the kidney in old age. Finally, we analyze the set of age-regulated genes to identify specific metabolic processes and cellular functions that change as a function of age, and discuss their possible roles in specifying the functional lifespan of the kidney.

## Results

**Figure 1:**
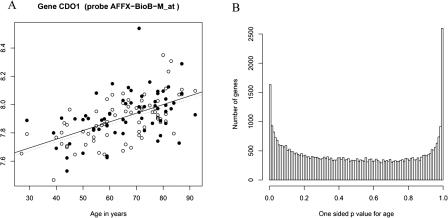
Age-Regulated Genes (A) Shown are expression levels for gene *CDO1*. White and black circles represent expression from cortex and medulla, respectively. The y-axis indicates log_2_ (expression level), and the x-axis indicates age of patient (years). Dotted and solid lines indicate best fit slopes for the cortex and medulla values, respectively. (B) For every gene, we calculated a one-sided p˜ -value that its expression changes with age. Shown is a histogram representing all of the genes represented by the Affymetrix DNA chip. Genes that decrease with age have p˜ -values near zero, and genes that increase with age have p˜ -values near one. If there were no age-regulated genes (i.e., the true *β_kj_* = 0 for every gene *j*), then the histogram of p˜ -values would be flat (i.e., have a uniform distribution on the interval from zero to one). The x-axis shows the p˜ -value, and the y-axis shows the number of genes with that p˜ -value. There are 985 genes with a *p-*value less than 0.001.

To procure material for analyzing changes in gene expression with age in the human kidney, we obtained kidney samples from normal tissue removed at nephrectomy for either removal of a tumor or for transplantation from 74 patients ranging in age from 27 to 92 y (Tables S1 and S2). We dissected each of the 74 kidney samples into cortex (72 samples) and medulla (62 samples) sections, isolated total RNA from each section, synthesized biotinylated complementary RNA (cRNA), and hybridized the labeled cRNA to Affymetrix high-density oligonucleotide arrays (HG-U133A and HG-U133B, containing a total of 44,928 probe sets corresponding to approximately 33,000 well-substantiated human genes). The level of expression for each gene was determined using DChip (Zhong et al. 2003), and the gene chip data were entered into the Stanford Microarray Database ( http://genome-www5.stanford.edu/) . Using our dataset, the expression level for every gene as a function of age could be plotted. For example, the expression of *CDO1* (which encodes a cysteine dioxygenase type 1 protein) tended to increase with age. There was also variation between subjects and between the cortex and the medulla (Figure 1A). Nearly all of the variation represents true differences between samples, as very little variation was observed when we performed repeat hybridizations using the same tissue sample (data not shown).

We used a linear regression model to identify genes that showed a statistically significant change in expression with age (i.e., were age-regulated). We saw large differences in expression between tissue types and between the sexes. These differences were of similar magnitude for both young and old subjects, so that aging in one tissue (or sex) typically ran parallel to aging in the other (as seen in Figure 1A). Our linear regression model allowed for these parallel trends; reasons for arriving at such a model are given below. Mathematically, our model takes the form



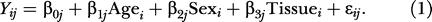



In equation 1, *Y_ij_* is the base 2 logarithm of the expression level for gene *j* in sample *i,* Age*_i_* is the age in years of the subject contributing sample *i,* Sex*_i_* is one if sample *i* came from a male subject (and zero for female), Tissue*_i_* is one if sample *i* was a medulla sample (and zero for cortex), and *ɛ_ij_* is a random error term. The coefficients *β_kj_* for *k* = 0, 1, 2, and 3 are values to be estimated from data. Our primary interest is in *β*_1*j*_ *,* which describes how quickly the expression of gene *j* changes with age, with*β*_1*j*_ = 0 for genes with no linear age relationship.

In model 1 and others that we considered, the coefficients were estimated by least squares. The estimated values β^_*kj*_ can differ from zero, even when the true coefficient is zero. We judged statistical significance through *p-*values, where a value of *p*_i*j*_ near zero corresponds to a large absolute value |β^_*kj*_| unlikely to have arisen by chance. Such *p-*values do not distinguish genes that increase with age from those that decrease with age. We also use one-tailed *p-*values, written p˜_*kj*_ *,* taking values near zero to be significantly decreasing trends and those near one to be significantly increasing trends (see Materials and Methods).

To make *p-*values comparable over genes, it is essential to use the same model for all genes. Before settling on the common model 1, we considered an alternative that allowed a quadratic trend in age. The p˜ -values for the quadratic coefficient (not shown) gave no reason to suspect that a curved relationship was needed. Similarly, a piecewise linear age relationship (with bends at ages 50 and 70) was not significantly better than a linear one. Large and statistically significant differences in expression were found for the two tissue types, and so the tissue type was included in equation 1. Incorporating tissue type into the model reduces the estimate of the noise variance, leading to greater power for detecting an age relationship. Similarly, a small number of genes were found to have significantly different expression between sexes. Seven genes were found to have a difference at *p <* 0.001 for both sex and age.

We performed a genome-wide scan for genes that changed expression with respect to age. Age-regulated genes can be identified by plotting p˜ -values for age based on model 1 (Figure 1B). Genes that significantly decrease in expression with age appear in a peak on the left, while those whose expression increases with age are in a peak at the right. Using model 1, we found 985 genes that change with respect to age (*p <* 0.001), which is considerably greater than would be expected by chance (approximately 45 from a total of 44,928 genes). Of these, 742 genes increase expression with age and 243 decrease expression with age (Table S3).

Most of our samples were taken from patients that underwent nephrectomy for various medical reasons (see Table S1). We evaluated whether pathology, medical history, or medication might be factors that confounded our aging analysis. For example, if old people tend to be hypertensive more often than young, then genes that respond to hypertension may appear to be age-related.

We identified 20 different medical and other factors that might potentially confound our study, including race, blood pressure, diabetes, and type and size of tumor present in the kidney (see Table S1). Fourteen factors (such as diabetes or proteinuria) affected less than ten patients, making it unlikely that they could account for age-related change in gene expression in the 74 patients analyzed. Six factors occurred in ten or more patients (non-white race, two types of tumors, size of tumor, and hypertension), but it is unlikely that these affected our aging study for the following reasons.

First, with the exception of transitional cell carcinoma, none of the other factors were skewed with respect to age, and would not be expected to bias gene expression in an age-related fashion (Figure S1).

Second, the two types of tumors (renal cell carcinoma and transitional cell carcinoma) were localized to an isolated region of the kidney. Our normal samples were obtained from the region of the kidney furthest from the carcinoma, were not directly contaminated with cancer cells, and appeared normal histologically (see Materials and Methods). This procedure for obtaining kidney samples has been used previously to profile gene expression in normal kidney (Higgins et al. 2004) and as a normal control in a kidney cancer study (Higgins et al. 2003).

Third, we used regression models to directly test whether our aging studies were affected by seven medical factors: renal cell carcinoma, transitional cell carcinoma, size of tumor, hypertension, systolic blood pressure, diastolic blood pressure, and diabetes mellitus. For renal cell carcinoma, we used a regression model predicting expression from age, sex, tissue type, and a zero/one variable indicating whether the sample came from a patient with renal cell carcinoma or not. The result gave a *p*-value for whether renal cell carcinoma affected each of the 44,928 genes present on the Affymetrix DNA chip. The smallest *p*-value was 0.00013. We would expect to see almost six such *p-*values by chance alone. This result indicates that the presence of renal cell carcinoma does not significantly affect the expression of any gene in the normal tissue from the same kidney, compared to normal tissues taken from kidneys without renal cell carcinoma.

Next, we plotted the results using only the age-regulated genes, to investigate whether adjustments for renal cell carcinoma could affect their change in expression with respect to age. We used one regression model that included a renal cell carcinoma term and another model that did not have the term. We then selected genes that showed statistically significant (*p <* 0.001) age regulation using either of these models. Renal cell carcinoma does not significantly affect the age slopes for these genes (Figure S2A), indicating that this medical factor has little effect on age-related gene expression.

We repeated the regression analysis for six other factors that might confound our results (transitional cell carcinoma, size of tumor, hypertension, systolic blood pressure, diastolic blood pressure, and diabetes mellitus). The regression slopes changed very little with and without these factors, indicating that these factors do not strongly affect our analysis of age regulation (Figure S2B–S2G).

Fourth, five of the samples were from kidneys that did not have tumors, and two of these were from donor kidneys used for transplantation that had no associated pathology at all. The expression profile from these five patients was similar to the profile from other samples used in our study. In summary, it is unlikely that these disease and medical factors have confounded our analysis of age-regulated changes in gene expression.

Changes in the expression for some of the 985 age-regulated genes may directly reflect the aging process in the kidney; these genes would serve both as aging markers and provide clues about molecular mechanisms for aging in the kidney. Other changes may result from an age-related change in the relative proportion of cell types within the kidney, such as would result from increased infiltration of immune cells with age. Finally, the expression changes may reflect the downstream response of the kidney to an age-related process elsewhere, such as would result from age-related changes in blood pressure or vascular supply.

### Common Mechanisms of Aging in the Cortex and Medulla of the Kidney

Since the cortex and medulla contain different cell types and have distinct functions, it was of interest to test whether they age similarly. It is possible that the pattern of degeneration in a particular cell type reflects those metabolic pathways that are used most heavily by that cell. For example, there could be deterioration in cell adhesion in glomerular epithelial cells that form part of the filtration barrier in the cortex, while there could be an age-related decline in ion traffic or water flow across the apical or basolateral membranes of tubular epithelial cells in the medulla. Alternatively, distinct cell types could show a common pattern of age-related decline involving pathways common to all cells, such as protein synthesis and mitochondrial function. This degeneration of core cellular processes would affect every cell function, including filtration by glomerular epithelial cells and water and solute reabsorption by tubular epithelial cells.

**Figure 2:**
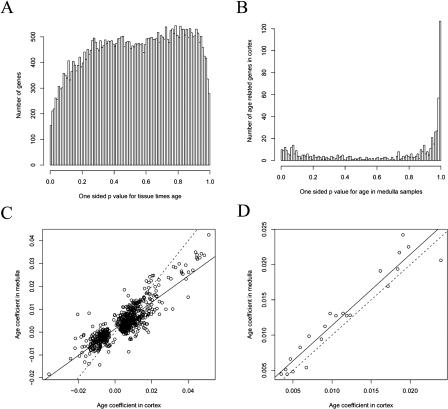
Similar Age-Regulation in Cortex and Medulla (A) For every gene, we calculated a p˜ -value that there is a Tissue*_i_* ×Age*_i_* effect, and plotted the results in a histogram. Genes that show different age regulation in the cortex or the medulla would be contained in peaks on the left and right parts of the histogram. The figure shows that the number of genes that have different expression levels in the cortex and medulla is about the same as or less than would be expected by chance. The x-axis shows one-sided p˜ -values for Tissue*_i_* ×Age*_i_,* and the y-axis shows the number of genes with that p˜ -value. There is a systematic under-representation of the edge regions compared to a random sample of uniform random variables because of correlations among the 44,928 p˜ -values computed from 133 samples. (B) To show whether aging in the cortex and the medulla is similar, we selected age-regulated genes in the cortex and calculated the one-tailed p˜ -value for age effects in the medulla. The histogram shows these selected p˜ -values. The spike at the right shows genes that increase with age in the medulla. Those genes also increased with age in the cortex. (C) Shown is a scatterplot of all 684 genes that are age-regulated in either the medulla or the cortex (*p <* 0.001). The y-axis is the slope for the medulla of the expression change with respect to age, and the x-axis is the slope for the cortex. The solid line is the least squares line, with a slope of 0.58. The dotted line has a slope of one and passes through the origin. (D) Same as (C) but for 22 genes that are age-regulated in both the cortex and the medulla (*p <* 0.001).

To test whether age-related gene expression changes are different in cortex and medulla, we considered a model in which a term of the form *β*_4*j*_ × Tissue × Age was added to the model in equation 1. In such a model, the change in expression with age is linear within each tissue type, but the slope in the medulla is larger than that in the cortex by*β*_4*j*_. Figure 2A shows the histogram of the p˜_4*j*_ -values. Genes showing tissue-specific slopes would appear in peaks on the left and right. The figure shows neither of these peaks, indicating there is no statistically significant difference in aging between the two tissue types.

To further investigate coordinate aging in the cortex and medulla, we searched for age-regulated genes in each of these tissues independently, and then tested whether age-regulated genes in one were also age-regulated in the other. Specifically, to find age-regulated genes in the cortex, we fit the model



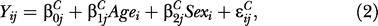



using the cortex samples only. To find age-regulated genes in the medulla, we fit the model



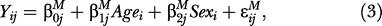



using only the medulla samples. We found 634 genes in the cortex samples and 72 genes in the medulla samples that showed significant changes in expression with age (*p <* 0.001).

Having identified age-regulated genes in the cortex, we next examined whether they were also age-regulated in the medulla. Figure 2B shows the p˜ -values for change with age in the medulla samples, for those genes that are age-regulated (*p* < 0.001) in the cortex samples. If aging in the medulla were unrelated to aging in the cortex, we would expect to see a flat histogram. The actual histogram has a strong peak of genes on the right, indicating that significantly age-regulated genes in the cortex tend to also be significantly age-regulated in the medulla. Of the 634 genes that increased expression with age in the cortex, 22 also increased expression with age in the medulla, compared with the 0.6 genes expected at *p =* 0.001. We obtained similar results when we took the converse approach, first selecting the 72 age-regulated genes in the medulla, and then testing whether they were also age-regulated in the cortex (data not shown).

Next, we compared the slope of expression with respect to age in the cortex to that in the medulla (Figure 2C). The results show a strong correlation between age coefficients in cortex and medulla. For the 684 genes age-regulated in at least one of the tissue types, the age coefficients had a correlation of *r =* 0.487. Models 2 and 3 allow us to investigate whether the cortex and medulla age at the same rate as specified in model 1. For the 22 genes that are significantly age-related in both tissues, the age coefficients have a high correlation (*r =* 0.96), and the slopes themselves are numerically close (Figure 2D). We found a small mean absolute difference in slopes of 0.00185 (log_2_ expression per year), corresponding to only a 6% divergence in expression over 50 y. Given the strong similarities in the aging profiles of these two tissue types, we are able to increase the statistical power of our analysis by pooling the cortex and medulla datasets (resulting in model 1).

### Increased Expression of Immune Genes in the Kidney in Old Age

We examined the list of 985 age-regulated genes, and immediately found evidence for increased expression of genes from immunocytes. Many of the 985 age-regulated genes are expressed specifically in B cells (e.g., immunoglobulin mu, kappa, and lambda), T cells (e.g., T cell receptor beta), or neutrophils (e.g., neutrophil cytosolic factors 1 and 4) (see Table S3). Nearly all of these immune genes increase expression with age. These results suggest that there are increased numbers of immune cells in the kidney in old age, resulting in an age-related increase in abundance in all genes that are expressed specifically in these cells. Immune function is known to decline with age, and the increased numbers of immunocytes in the kidney might compensate for decreased function in individual immune cells, either for immune surveillance or for responding to low levels of inflammation occurring normally. In addition to increased cell numbers, the apparent increase in expression of the immune genes could also be due to increased expression within the immune cells themselves.

Immunohistochemical experiments using antibodies directed against markers specific for B cells, T cells, or neutrophils showed that the kidney samples contained a small proportion of immune cells (less than 1%) in sporadic clusters scattered throughout each section (data not shown). The number of immune cells varied greatly from section to section, and thus it was not possible to use immunohistochemistry to confirm a quantitative increase in the numbers of immune cells in the kidney with age.

**Figure 3:**
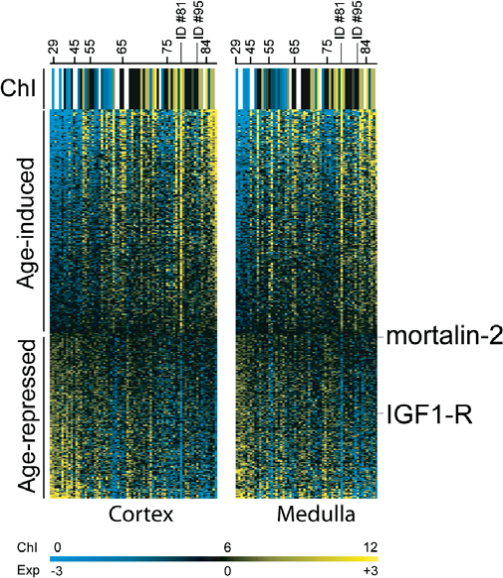
Expression of the 447 Genes as a Function of Age Rows correspond to age-regulated genes, ordered from most highly induced to most highly repressed. Columns correspond to individual patients, ordered from youngest to oldest. The age of certain patients is shown for reference. Left panel refers to data from cortex samples, and right panel depicts data from medulla samples. The first row shows the chronicity index (ChI; morphological appearance and physiological state of the kidney),from blue (healthiest) to yellow (least healthy) as indicated in the scale bar. Key genes discussed in the text are marked. Scale shows log_2_ of the expression level (Exp). A navigable version of this figure can be found at http://cmgm.stanford.edu/~kimlab/aging_kidney/explorer.html.

If the number of immune cells increases with age in our kidney samples, then any gene showing an age-related increase in expression might do so because it is expressed in immune cells and not because it is age-regulated in the kidney. As immune cells comprise only a small fraction of the kidney sample, age-regulated genes that are expressed at higher levels in the kidney than the blood are likely to be expressed in kidney cells themselves. To compare gene expression levels between the blood and the kidney, we purified RNA from whole blood from five new individuals, prepared labeled cRNA, and then hybridized it to Affymetrix gene chips in the same manner as before. We computed the log_2_ of the expression level for each gene, and then calculated an average expression level for the blood (five samples) and the kidney (134 samples). Of the 985 genes that change expression with age, 538 are expressed at higher levels in blood cells than in the kidney samples. Age-related changes in the RNA abundance of these genes may reflect either changes in the fraction of immune cells in the kidney or age-related changes in expression in kidney cells. The remaining 447 are expressed at higher levels in the kidney than in whole blood, and age regulation of these genes is likely to reflect expression changes in kidney cells themselves (Table S4). Of these 447 genes, 257 have increased expression levels in old age (age-induced) and 190 have decreased expression levels (age-repressed) (Figure 3).

### Age Regulation Compared to Developmental Regulation

**Figure 4:**
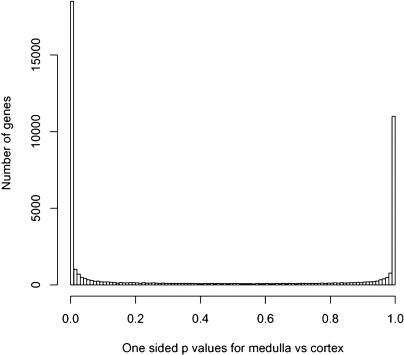
Differential Expression in the Cortex and the Medulla For each gene, we calculated a p˜ -value for expression differences in the cortex versus the medulla. Shown is a histogram of these p˜ -values. Genes enriched in the cortex are in a peak on the left, and genes enriched in the medulla are in a peak on the right. The x-axis indicates p˜ -value, and the y-axis indicates number of genes.

Aging is thought to be caused by slow degeneration of the transcriptome (the entire set of genes expressed in a tissue), rather than a qualitative change in expression, as occurs during tissue specification. As such, changes in gene expression associated with aging should be less than expression differences between different types of tissues. To confirm this idea, we compared the magnitude of gene expression differences due to differentiation (cortex versus medulla) to those due to aging. We used the same approach as before to evaluate differences in expression in cortex versus medulla on a genome-wide scale. For every gene, we calculated the *p-*value for differential expression in the cortex and the medulla, and plotted the results in a histogram (Figure 4). Genes contained in the peak on the right are more abundant in the medulla whereas genes in the peak on the left are more abundant in the cortex. There were 23,322 genes that were differentially expressed between the cortex and medulla (*p <* 0.001), indicating that regulation of expression due to differentiation (between the cortex and medulla) is much greater than that related to aging. This result is consistent with the idea that aging results from a slow degeneration of a core transcriptome in the cortex and the medulla of the kidney.

### Majority of Age-Regulated Genes in the Kidney Are Expressed Broadly

To address whether different organs have distinct or common aging profiles, we analyzed whether the 447 age-regulated genes in the kidney were expressed specifically in the kidney or broadly in many tissues. If the kidney has its own specific pattern of aging, one might expect that the set of 447 aging-regulated genes would be enriched for those expressed specifically in the kidney, such as genes that have direct roles in forming the filtration barrier or in regulating ion or water reabsorption. If there is a common profile for aging shared among tissues, one might expect that most of the list of 447 aging-regulated genes would be expressed in many tissues.

**Figure 5:**
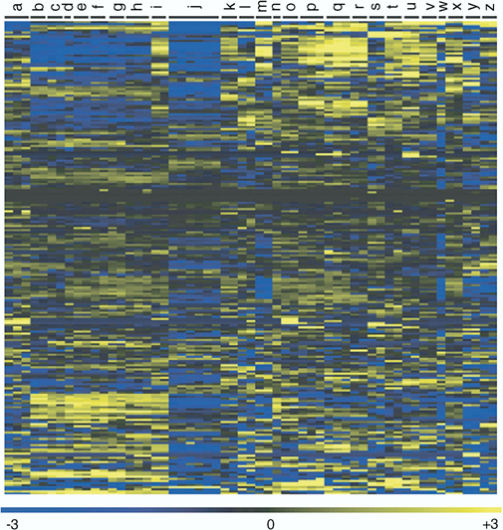
Developmental Profile of the Age-Regulated Genes Shown are the log_2_ of the expression levels for 227 age-regulated genes in 26 human tissues, using data from Su et al. (2002). Rows correspond to genes, columns correspond to human tissues. a, kidney; b, cerebellum; c, whole brain; d, cerebral cortex; e, caudate nucleus; f, amygdala; g, thalamus; h, corpus callosum; i, spinal cord; j, whole blood; k, testis; l, pancreas; m, placenta; n, pituitary gland; o, thyroid gland; p, prostate; q, ovary; r, uterus; s, salivary gland; t, trachea; u, lung; v, thymus; w, spleen; x, adrenal gland; y, liver; z, heart. Scale shows log_2_ of the expression level. A navigable version of this figure can be found at http://cmgm.stanford.edu/~kimlab/aging_kidney/explorer.html.

We determined the level of expression of the age-regulated genes in different tissues using data from a previous study reporting a genome-wide profile of gene expression in 26 different human tissues with Affymetrix gene arrays (Su et al. 2002). Of the 447 aging-regulated kidney genes, 227 are represented in the previous work. Nearly all of these have general, rather than kidney-specific, expression patterns; specifically, we calculated the median expression level from all tissues and compared this to the average expression level from the kidney samples. We found that only seven of the 227 aging-regulated genes were enriched in the kidney more than 2-fold compared to the median level from all tissues (Figure 5). The observation that nearly all of these 227 age-regulated genes are expressed in many tissues suggests that they act in common cellular pathways. Altered expression of these genes in old age may weaken these common functions, subsequently leading to physiological decline of kidney-specific functions.

### Molecular Markers for Physiological Aging

The expression levels of these 447 age-regulated genes constitute a molecular profile of aging, and we can examine the expression profile of individual patients to observe how they compare to the average for their age group. Older individuals tended to express age-induced genes at higher levels and age-repressed genes at lower levels than younger individuals. However, certain individuals had unusual expression profiles, in which genes were expressed at levels more typical of a different age group. For example, patient 81 was 78 y old but had an expression profile as though she were older (see Figure 3). Her kidney showed very high levels of age-induced genes and very low levels of age-repressed genes. Patient 95 was 81 y old, with an expression profile similar to patients 30 or 40 y younger.

**Figure 6:**
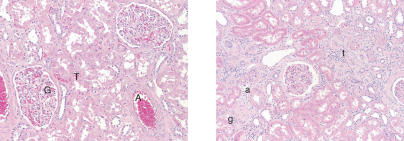
Chronicity Index of Kidney Samples Histology from patient 40 is shown on the left, demonstrating a normal glomerulus (G), tubules and interstitial space (T), and arteriole (A), respectively (chronicity score of zero). Histology from patient 62 is shown on the right, demonstrating glomerulosclerosis (g), tubular atrophy and interstitial fibrosis (t), and arterial intimal hyalinosis (a), respectively (chronicity score of ten). Hematoxylin and eosin staining of paraffin-embedded sections.

**Figure 7:**
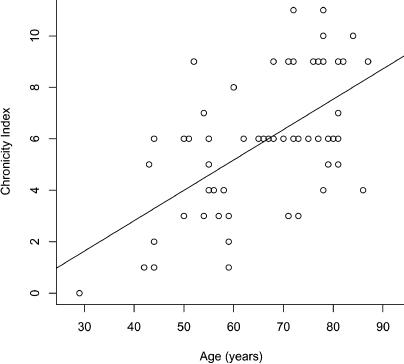
Chronicity Index Increases with Age Shown is the chronicity index versus age for most of the kidney samples used in this study. The line shows the least squared fit through the data points.

Do the molecular gene expression profiles correlate with the physiological ages of the kidney samples? That is, does patient 81 have a kidney showing excessive age-related damage and does patient 95 have a kidney with unusually good health? To answer these questions, we determined the morphological and physiological states of the kidneys from each of the patients by examining histological stains. As people grow older, there is a general decline in the morphological appearance of the kidney: (1) the glomeruli lose their structure and their capillaries are replaced with fibrous tissue (glomerular sclerosis), (2) the tubules collapse and atrophy, and the interstitial space between them widens and scars (tubular atrophy/interstitial fibrosis), and (3) there is a thickening of the innermost layer of the arteriole wall due to the accumulation of hyaline material (arterial intimal hyalinosis). We gave three scores to each kidney section corresponding to the appearance of the glomeruli, the tubules, and the arterioles. Scores ranged from zero for normal appearance for youthful patients to four for an advanced state of glomerular sclerosis, tubular atrophy/interstitial fibrosis, or arterial intimal hyalinosis (see Table S1). We then added the glomerular, tubular, and arteriolar scores together to form a combined score ranging from zero (best) to 12 (worst), termed the chronicity index. The chronicity index is a quantitative estimate of the morphological appearance and physiological state of the kidney for each of the patients (see Table S1). Figure 6 shows an example of a kidney in good condition from patient 40 (29 y old with a chronicity score of zero) and a kidney showing age-related morphological decline from patient 62 (84 y old with a chronicity score of ten). As expected, the chronicity index shows a strong positive correlation with age showing that morphology and function tend to be worse for older subjects (Figure 7).

We then compared the chronicity index to the gene expression profiles of the 447 age-regulated genes as a function of age (see Figure 3). In general, we found that the gene expression profiles correlated well with the chronicity index. Patients with expression profiles normally associated with people much older also had a high chronicity index; for example, the expression profile of patient 81 was similar to that of patients who were much older, and the chronicity index was also unusually high for the patient's age. Conversely, patients with expression profiles normally associated with younger people tended to have a low chronicity index for their age, such as patient 95. Although the 447 age-regulated genes were selected solely on the basis of their change with chronological age, these results indicate that their expression profiles are able to predict patients that have kidneys exhibiting unusual health or abnormal degeneration for their age. Thus, the 447 age-regulated genes can be used as molecular markers for physiological decline in the kidney during aging.

### Age-Regulated Genes in the Kidney

Some of the 447 age-regulated genes may be involved in either causing or preventing aging in the kidney, whereas expression changes for others may be a consequence of age-related cellular changes. A candidate from our list that might promote age-related decline is *mortalin-2* (which encodes Heat Shock Protein 70), which decreases expression in the kidney in old age. Heat shock proteins act as protein chaperones, and likely function to counteract cell senescence by alleviating the accumulation of damaged proteins in old cells. In human fibroblasts, overexpression of *mortalin-2* extends lifespan in vitro (Kaul et al. 2003). In the nematode *C. elegans,* overexpression of mortalin or HSP-16 (a related heat shock protein) extends longevity, and several genes encoding heat shock proteins decrease expression in old age (Lund et al. 2002). Reduced expression of *mortalin-2* in old human kidneys could increase the accumulation of denatured proteins and thereby reduce general cellular function.

A gene from our list that might function to prevent aging is the gene encoding insulin-like growth factor receptor, which decreases expression in old age. Loss-of-function mutations in this gene result in extended longevity in worms, flies, and mice (Tissenbaum and Guarente 2002). This observation suggests that decreased expression of this gene during normal aging might help prolong the functional lifespan of human kidneys.

We examined the list of 447 age-regulated genes for functional groups showing a consistent change with age. One group includes genes involved in the formation of the extracellular matrix, which show a consistent increase in expression in old age. Seven age-regulated genes encode proteins known to play key roles in maintaining epithelial polarity (three types of claudins, two cadherins, occludin, and a cell adhesion molecule), all but one of which increase expression in old age (see Table S4). Forty-nine age-regulated genes encode protein components of the extracellular matrix, all but four of which increase expression in old age. In the kidney, the extracellular matrix could play a key role in governing the filtration of blood via the basement membrane, a capacity that declines with age. The observation that genes involved in forming the extracellular matrix increase expression in the kidney with age may be directly relevant to the age-related decline in glomerular filtration rate.

Another functional group is a set of 11 genes encoding ribosomal proteins, all of which increase expression with age. Protein synthesis rates are known to decline as animals grow older, and increased expression of these ribosomal protein genes may serve to offset this.

Changes in the expression of regulatory genes with age may have particularly strong effects on kidney metabolism and function, since these changes are likely to initiate cascades of changes in downstream genes. We examined our list of 447 age-regulated genes for those that are likely to function as regulatory genes. Of the 447 age-regulated genes, 15 encode transcription factors and 51 encode proteins that are part of signaling pathways.

### Age-Regulated Genes Enriched in the Glomeruli

As filtration of the blood takes place in the glomerulus, age-regulated genes that are enriched in the glomerulus may be especially important for understanding how kidney function declines with age. We identified genes enriched in the glomerulus using data from a previous study, in which cDNA microarrays were used to compare expression levels in the glomeruli relative to the rest of the kidney (Higgins et al. 2004). Of the 447 genes identified in our study, 213 were represented on the cDNA microarrays in the previous experiment, and 19 were enriched greater than 2-fold in the glomeruli relative to total kidney (Table S5). These included four genes that encode proteins involved in the formation of the extracellular membrane (a type 5 collagen, alpha-2 macroglobulin, and two tissue inhibitors of met-alloproteinase), all of which increase expression with age.

## Discussion

Old age is associated with a functional decline in a myriad of molecular and cellular processes. To gain a global perspective of the diverse pathways that change with age, we performed a whole-genome analysis of gene expression as a function of age for kidney samples from 74 patients ranging in age from 27 to 92 y. Many factors affect gene expression in addition to age, including variability between individuals, between different tissues within the kidney, and between sexes. The large number of samples in our dataset provided good power for identifying age-regulated genes in noisy data despite small changes in expression, and allowed us to use a statistical linear regression model to identify 985 genes that change expression with age.

The results from this work show that transcriptional differences between young and old individuals involve an accumulation of small changes in expression from many genes, rather than resulting from large expression changes in a small number of genes. This observation suggests that functional decline in old age is not the result of the complete failure of a small number of cellular processes. Rather, it is the slight weakening of many pathways that cumulatively causes a significant decrease in cell function. Studying aging by analyzing one pathway at a time is difficult, because any single pathway might show only a small change with respect to age and might contribute only a small amount to the overall functional decline in old age. By contrast, functional genomics is a powerful approach to study aging, because many genes can be simultaneously scanned in parallel for small changes in expression.

Although the cortex and medulla are comprised of different types of cells and perform different physiological functions, our results suggest that they share a common mechanism for aging. Previous experiments have characterized changes in expression for human fibroblasts, muscle, and the retina with age (Ly et al. 2000; Yoshida et al. 2002; Welle et al. 2003, 2004). We plotted the expression levels of the 985 aging-regulated genes found in this work against the dataset of aging in muscle (Welle et al. 2003), and found that these genes did not show much age regulation in muscle. Specifically, the Pearson correlation *(r)* of the regression slopes for these 985 genes was only 0.085 between the kidney and muscle aging experiments and hence accounts for only 0.0072 of the variance between these two tissues (Figure S3). It is unclear whether this amount of correlation is biologically relevant. The small sample size used in the study of aging in human muscles might have limited our ability to detect similarities in aging in the two organs. It will be important to use a larger sample size of muscle tissues in future experiments to discern common patterns of age regulation in the kidney and the muscle with higher resolution.

Aging has been best studied in model organisms, and it is thus of great interest to discern whether aging in these species is similar to the aging process in humans. Previous studies have reported gene expression changes associated with old age for worms, flies, and several tissues from mice (Lee et al. 1999, 2000; Hill et al. 2000; Zou et al. 2000; Lund et al. 2002; Pletcher et al. 2002; Murphy et al. 2003). We found no correlation between age regulation in human kidney and age regulation in either worms or flies (Figure S4).

Although our analysis did not show evidence for evolutionary conservation of age regulation, a previous study suggested that there is a small overlap in age-regulated gene expression between flies and worms (McCarroll et al. 2004). However, most of the similarities occured in young or middle-aged animals, rather than old animals. There is thus little evidence for evolutionary conservation of changes in gene expression in old age, emphasizing the need to elucidate mechanisms of aging using human subjects themselves and not model organisms.

Many of the age-regulated genes in the kidney may change in response to declining kidney function. Functional decline of the kidney with age varies between individuals, and these genes could be used as diagnostic markers to evaluate levels of kidney function in older patients. This could provide invaluable information in understanding the clinical course of kidney aging and the suitability of using older kidneys in organ transplants. Other genes may be directly regulated by aging per se, and these genes could pinpoint mechanisms that play key roles in the aging process itself.

## Materials and Methods

### 

#### Samples

Normal kidney samples were obtained either from biopsies of donor kidneys for transplantation or from nephrectromy patients (with informed consent) in which the pathology was localized and did not involve the part of the kidney sampled. Key factors from the medical record for each patient used in this study are listed in Table S1, and include sex, race, age, blood pressure, pathology, medications, serum creatinine, and urinary protein concentrations. Kidney tissue was harvested meticulously with the intention of gathering normal tissue uninvolved in the tumor. Tissue was taken from a point as far away from the tumor as possible. Any samples that showed evidence of pathological involvement or in which there was only tissue in close proximity to the tumor were discarded. Kidney sections were immediately frozen on dry ice and stored at −80 °C until use. The same harvesting sources and techniques have been used previously to profile expression in normal kidney (Higgins et al. 2004) and to provide normal controls in a study on kidney cancer (Higgins et al. 2003).

#### Histology

Frozen tissues were placed in cryomolds, embedded in Cryo Tissue Tek O.C.T. Compound (Sakura Finetek, Torrance, California, United States) and cut into 4-μm sections (Leica Microsystems, Wetzlar, Germany). Sections were stained with hematoxylin and eosin, and then histologically evaluated to exclude samples showing abnormal histology. Histology slides were also marked into two main functional sections, the cortex and medulla, to help aid in accurate dissection of these two areas. We reviewed radiological findings for all tumors and histology for all slides. We excluded any cases in which radiological imaging, gross examination at the time of resection, or histological review of the removed tissue indicated that there might be tumor involvement of the normal areas. Cases with incomplete or unclear medical records were excluded from this study.

#### RNA isolation

Frozen kidney tissue samples were dissected into cortex and medulla sections. Portions were weighed (0.05–0.75 g), cut into small pieces on dry ice, and then placed in 1 ml of TRIzol Reagent (Invitrogen, Carlsbad, California, United States) per 50–100 mg of tissue. The tissue was homogenized using a PowerGen700 homogenizer (Fisher Scientific, Pittsburgh, Pennsylvania, United States), and the total RNA was isolated according to the TRIzol Reagent protocol.

#### High-density oligonucleotide arrays

A standard protocol designed by Affymetrix (Santa Clara, California, United States) for their HG-U133A and HG-U133B high-density oligonucleotide arrays was slightly modified by the Stanford Genome Technology Center (Stanford, California, United States), and all samples were processed in their facility (see Protocol S1). Eight micrograms of total RNA was used to synthesize cRNA for each sample, and 15 μg of cRNA was hybridized to each DNA chip. The samples were done in random order with respect to tissue type and age.

#### Microarray data normalization and analysis

Using the dChip program (Zhong et al. 2003), microarray data (.cel files) were normalized according to the stable invariant set, and gene expression values were calculated using a perfect match model. All arrays passed the quality controls set by dChip. All of the Affymetrix data are available at the Stanford Microarray Database ( http://genome-www5.stanford.edu/) and at the Web site http://cmgm.stanford.edu/approximatelykimlab/aging_kidney/. The Affymetrix probe IDs and the locus link IDs for the genes discussed in the paper are in Tables S3–S5. The accession numbers for all genes on the Affymetrix arrays can be obtained from the Stanford Microarray Database.

#### Regression models and *p-*values

The *p-*values we use are based on *t*-tests from standard linear regression theory. Under the hypothesis H_0_ that *β_kj_* = 0, the estimated coefficient β^_*kj*_ is a random variable. The least squares value is a particular number, β^^*LS*^_*kj*_ . The *p-*value measures the extent to which the least squares value is surprisingly large assuming H_0_ holds. Specifically, the two-tailed *p-*value is



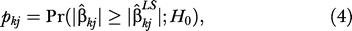



and the one-tailed *p-*value we use is



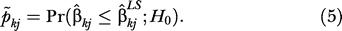



Sometimes p˜_*kj*_ is employed to test H_0_ against an alternative hypothesis of *β_kj_* < 0. We use it because it distinguishes between significant increasing and significant decreasing coefficients. Under H_0_, the distribution of *p* is *U*(0,1), and so is that of p˜ . Numerically, the equation



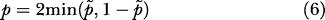



holds.

The *t*-test is derived under an assumption of normally distributed errors. The data showed estimated errors with heavier than normal tails. The *t*-test is known to be robust against heavy-tailed errors.

A linear regression is more appropriate for these data than is an analysis of variance (ANOVA) on age groups, because the latter is aimed at piecewise constant expression patterns, and it is not plausible that expression should change sharply at a given age. A genome-wide ANOVA (data not shown) did, however, find a similar group of age-related genes. Unlike ANOVA, regression summarizes the age effect in one coefficient. This is advantageous for interpretation and for statistical power when there is little nonlinearity.

The decision of whether to include a variable in model 1 was based on the collection of *p-*values for all the genes. If the histogram of p˜ values differed sharply from uniform, and if the smallest *p-*values were small compared to 1/44,928, then the coefficient was included.

Gene lists were made using a threshold *p-*value of 0.001. Such a gene list can be expected to have about 44 genes in it by chance, even if all of the coefficients are really zero. Thus, of the 985 age-related genes, it is plausible that about 44 of them are false positives. We have chosen to work with a fixed significance level, instead of attempting to fix the false discovery rate, because our test statistics are strongly correlated.

We were concerned that intra-subject correlations might have affected our results. For each of 59 subjects with both cortex and medulla samples, we subtracted log_2_ expression in the cortex from that in the medulla, and fit a regression of the difference versus age and sex. Such an analysis removes intra-subject correlations. There was again no evidence of genes aging differently in the two tissue types (data not shown).

## Supporting Information

Figure S1 Age Distribution of Medical and Related Factors Each row shows the presence of a medical or related factor. Age of patients is shown on the y-axis. Only transitional cell carcinoma showed a strong age bias. We have identified over 20 different factors that might potentially confound our study on aging, such as race, blood pressure, diabetes, and type and size of tumor adjacent to the normal section (see Table S1).
(221 KB PDF).

Figure S2 Medical Factors Do Not Affect Age Regulation We used regression models to directly test whether our aging studies were affected by seven medical factors: renal cell carcinoma, transitional cell carcinoma, size of tumor, hypertension, systolic blood pressure, diastolic blood pressure, or diabetes mellitus. Scatterplots show age-related slopes using a regression model that includes a term for the medical factor compared to slopes from a regression model that does not include that medical factor.
(A) Effect of renal cell carcinoma (RCC) on age-related expression. We selected genes that showed statistically significant (*p <* 0.001) age regulation using either a model with a renal cell carcinoma term or without a renal cell carcinoma term. The vertical and horizontal axes show the slope from a model with and without the renal cell carcinoma term, respectively. The slopes change very little with and without the renal cell carcinoma term. As one might expect, many of the genes that are significant at the 0.001 level are just barely so. There were 866 genes significant in both models, 119 significant only when renal cell carcinoma was not in the model, and 86 significant only when renal cell carcinoma was in the model. The overall picture of age relationship changes very little whether a term for renal cell carcinoma is included in the model or not.
We also used a regression model predicting expression from age, sex, tissue type, and a zero/one variable indicating whether the sample came from a patient with renal cell carcinoma or not. The result gave a *p-*value for whether renal cell carcinoma affected each of the 44,928 genes present on the Affymetrix DNA chip. The smallest *p-*value we saw was 0.00013. We would expect to see almost six such *p-*values by chance alone. This result indicates that the presence of renal cell carcinoma does not significantly affect the expression of any gene in the normal tissue from the same kidney, compared to normal tissues taken from kidneys without renal cell carcinoma.
(B) Effect of transitional cell carcinoma (TCC) on age-related expression. Scatterplot showing age-related slopes with and without a term for transitional cell carcinoma. Transitional cell carcinoma was present in 13 patients, all of whom were old. Thus if transitional cell carcinoma affected gene expression in adjacent normal tissue, then it might bias our results on aging. (B) shows data for presence or absence of transitional cell carcinoma in the model. The gene with the smallest *p-*value for transitional cell carcinoma had a *p-*value of 8.8 × 10^−6^. The expected number of *p-*values this small in 44,928 trials is 0.4, so the presence of this gene is not particularly compelling evidence that transitional cell carcinoma biased our results. The histogram of *p-*values looks uniform, as we would expect if transitional cell carcinoma were very weakly related, or not related, to expression changes with age (data not shown). We have not used false discovery rate techniques for this problem, because the age coefficients for different genes are far from independent. The scatterplot shows that transitional cell carcinoma does not affect age-related slopes very much.
(C) Tumor size does not affect age regulation.
(D) Hypertension (HTN) does not affect age regulation.
(E) Systolic blood pressure (SBP) does not affect age regulation.
(F) Diastolic blood pressure (DPB) does not affect age regulation.
(G) Diabetes mellitus (DM) does not affect age regulation.
(307 KB JPG).

Figure S3 Comparison of Age Regulation of Gene Expression between Kidney and Muscle Tissue in Humans We obtained the muscle dataset from the GEO database (Welle et al. 2003). To compare age regulation in the kidney and muscle, we queried whether the 447 genes identified as age-regulated in the kidney were similarly age-regulated in the muscle. We determined regression coefficients for the 447 genes in the muscle dataset using multiple regression, in a manner similar to the kidney dataset. For each of the 447 genes, we plotted regression slope in kidney against regression slope in muscle, and found an overall weak Pearson correlation of 0.085 (*p* < 0.004). A Pearson correlation value of 0.085 implies that 0.72% of the variance in the muscle regression coefficients is due to variance in the associated kidney regression coefficients. We note that the muscle dataset had a small sample size (*n* = 16), which may not be large enough to sufficiently detect similarity in age regulation with the kidney.
(59 KB XLS).

Figure S4 Comparison of Age Regulation of Gene Expression between Humans, Flies, and Worms Reveals No Correlation We compared patterns of gene expression in the aging time course data from C. elegans (Lund et al. 2002) and D. melanogaster (Pletcher et al. 2002) to those in the data for the human kidney. We identified orthologous genes using the criterion that they exhibit best reciprocal BLAST hits between species. Beginning with the set of 447 age-regulated genes in the human kidney, we identified 119 worm and 142 fly orthologs. From the set of 167 age-regulated genes in the worm, we identified 60 human orthologs. From 1,264 age-regulated genes in the fly, we identified 465 human orthologs.
(A) Regression slopes of age-regulated genes from human kidney and D. melanogaster. Open triangles denote age-regulated genes in humans and their orthologs in flies. Open circles denote age-regulated genes in flies and their orthologs in humans. The scatterplot shows the regression slopes from the human kidney and the fly aging datasets (Pletcher et al. 2002). Specifically, the age-regulated human genes paired with fly orthologs show a Pearson correlation *r =* −0.05 (*p =* 0.27) for human and fly, while the age-regulated fly genes paired with human orthologs show a Pearson correlation *r =* −0.05 (*p =* 0.12).
(B) Regression slopes of age-regulated genes from human kidney and C. elegans. Open circles denote age-regulated genes in humans and their orthologs in worms. Open triangles denote age-regulated genes in worms and their orthologs in humans. The scatterplot shows the regression slopes from the human kidney and C. elegans aging datasets (Lund et al. 2002). The age-regulated human genes paired with worm orthologs show a Pearson correlation *r =* 0.05 (*p =* 0.54). The age-regulated worm genes paired with human orthologs show a Pearson correlation *r =* −0.01 (*p =* 0.08).
These results show no evidence for overlap in the aging process between different species.
(509 KB PDF).

Protocol S1 Affymetrix HG-U133 Set Gene Chip Protocol (40 KB DOC).

Table S1 Medical History of Patients (33 KB XLS).

Table S2 Patients Recruited by Age Group (13 KB XLS).

Table S3 Age-Related Genes (*p <* 0.001) Arranged by *p-*Value (135 KB XLS).

Table S4 Age-Related Genes (*p <* 0.001) Excluding Those with Higher Expression Levels in Blood than in Kidney, Arranged by Fold Change (75 KB XLS).

Table S5 Age-Related Genes by Location within the Kidney (53 KB XLS).

## Abbreviations

ANOVA: analysis of variance
cRNA: complementary RNA
