# Supplementary figures and images for: A Transcriptional Profile of Aging in the Human Kidney
Source: PLoS Biol. 2004 Nov 30;2(12):e427. doi: 10.1371/journal.pbio.0020427 (PMC532391; doi:10.1371/journal.pbio.0020427)

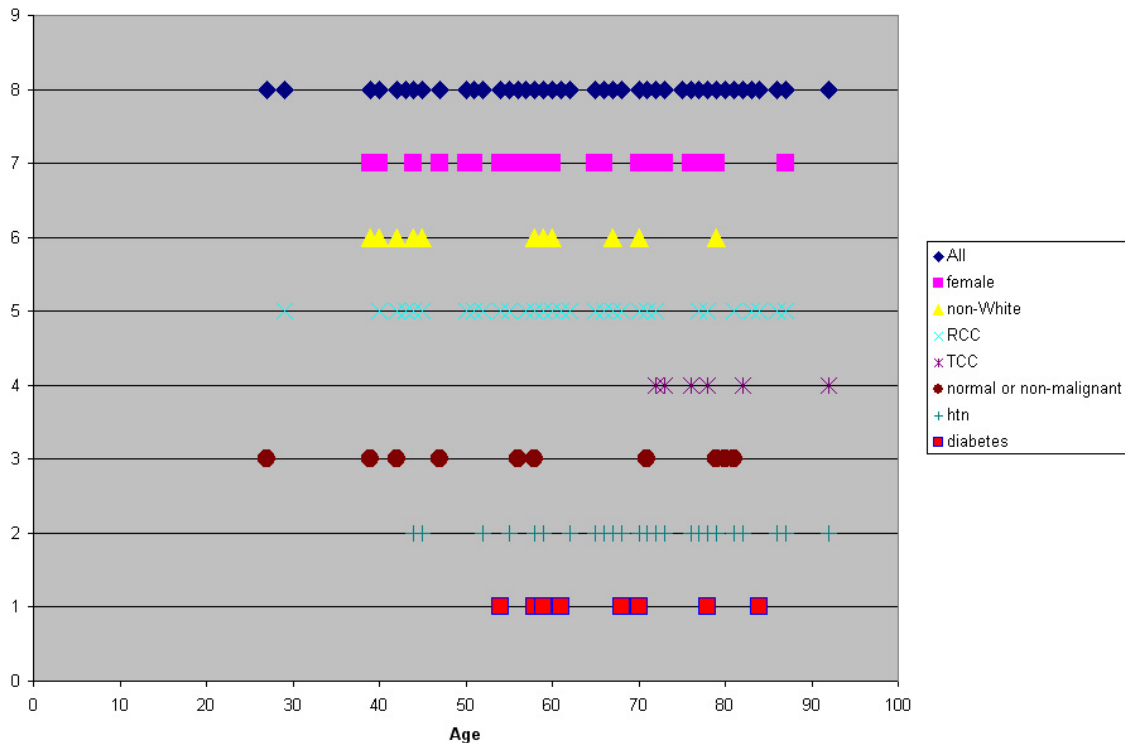

Supplement: Figure S1 — Each row shows the presence of a medical or related factor. Age of patients is shown on the y-axis. Only transitional cell carcinoma showed a strong age bias. We have identified over 20 different factors that might potentially confound our study on aging, such as race, blood pressure, diabetes, and type and size of tumor adjacent to the normal section (see Table S1). (221 KB PDF). [file pbio.0020427.sg001.pdf]

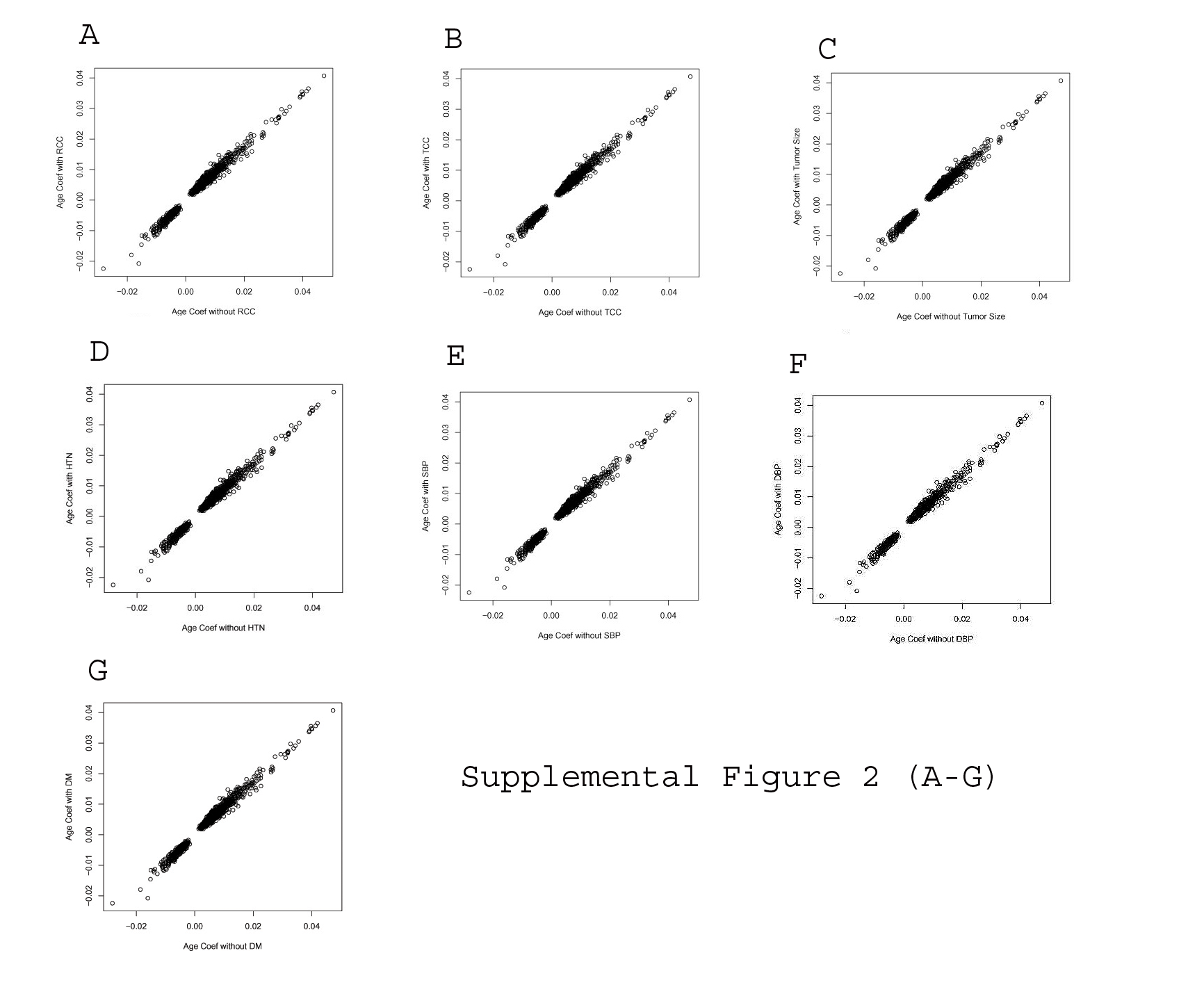

Supplement: Figure S2 — We used regression models to directly test whether our aging studies were affected by seven medical factors: renal cell carcinoma, transitional cell carcinoma, size of tumor, hypertension, systolic blood pressure, diastolic blood pressure, or diabetes mellitus. Scatterplots show age-related slopes using a regression model that includes a term for the medical factor compared to slopes from a regression model that does not include that medical factor. (A) Effect of renal cell carcinoma (RCC) on age-related expression. We selected genes that showed statistically significant (p < 0.001) age regulation using either a model with a renal cell carcinoma term or without a renal cell carcinoma term. The vertical and horizontal axes show the slope from a model with and without the renal cell carcinoma term, respectively. The slopes change very little with and without the renal cell carcinoma term. As one might expect, many of the genes that are significant at the 0.001 level are just barely so. There were 866 genes significant in both models, 119 significant only when renal cell carcinoma was not in the model, and 86 significant only when renal cell carcinoma was in the model. The overall picture of age relationship changes very little whether a term for renal cell carcinoma is included in the model or not. We also used a regression model predicting expression from age, sex, tissue type, and a zero/one variable indicating whether the sample came from a patient with renal cell carcinoma or not. The result gave a p-value for whether renal cell carcinoma affected each of the 44,928 genes present on the Affymetrix DNA chip. The smallest p-value we saw was 0.00013. We would expect to see almost six such p-values by chance alone. This result indicates that the presence of renal cell carcinoma does not significantly affect the expression of any gene in the normal tissue from the same kidney, compared to normal tissues taken from kidneys without renal cell carcinoma. (B) Effect of tra [file pbio.0020427.sg002.jpg]

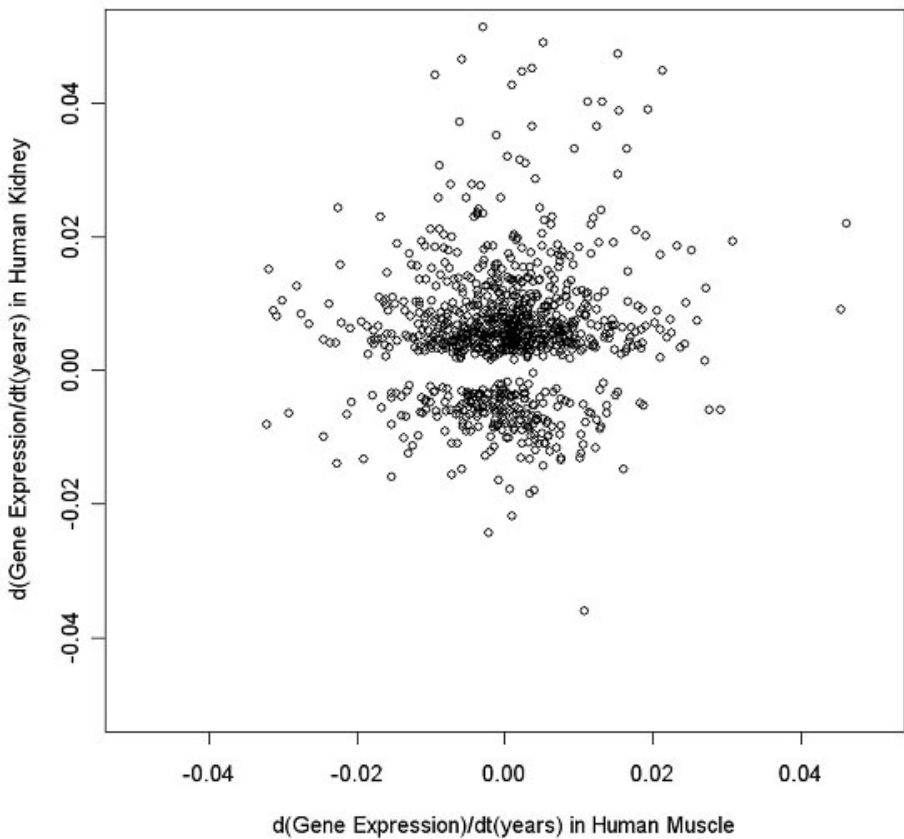

Supplement: Figure S3 — We obtained the muscle dataset from the GEO database (Welle et al. 2003). To compare age regulation in the kidney and muscle, we queried whether the 447 genes identified as age-regulated in the kidney were similarly age-regulated in the muscle. We determined regression coefficients for the 447 genes in the muscle dataset using multiple regression, in a manner similar to the kidney dataset. For each of the 447 genes, we plotted regression slope in kidney against regression slope in muscle, and found an overall weak Pearson correlation of 0.085 (p < 0.004). A Pearson correlation value of 0.085 implies that 0.72% of the variance in the muscle regression coefficients is due to variance in the associated kidney regression coefficients. We note that the muscle dataset had a small sample size (n = 16), which may not be large enough to sufficiently detect similarity in age regulation with the kidney. (59 KB XLS). [file pbio.0020427.sg003.xls]

A

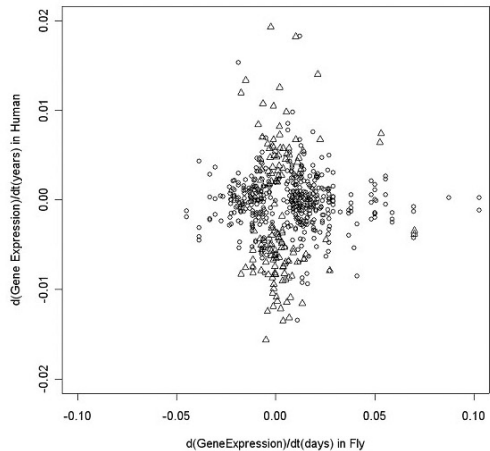

B

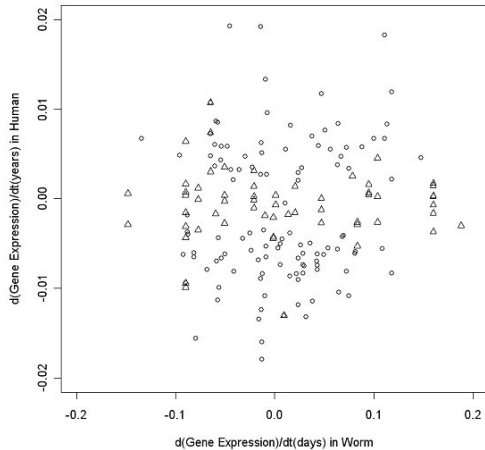

Supplement: Figure S4 — We compared patterns of gene expression in the aging time course data from C. elegans (Lund et al. 2002) and D. melanogaster (Pletcher et al. 2002) to those in the data for the human kidney. We identified orthologous genes using the criterion that they exhibit best reciprocal BLAST hits between species. Beginning with the set of 447 age-regulated genes in the human kidney, we identified 119 worm and 142 fly orthologs. From the set of 167 age-regulated genes in the worm, we identified 60 human orthologs. From 1,264 age-regulated genes in the fly, we identified 465 human orthologs. (A) Regression slopes of age-regulated genes from human kidney and D. melanogaster. Open triangles denote age-regulated genes in humans and their orthologs in flies. Open circles denote age-regulated genes in flies and their orthologs in humans. The scatterplot shows the regression slopes from the human kidney and the fly aging datasets (Pletcher et al. 2002). Specifically, the age-regulated human genes paired with fly orthologs show a Pearson correlation r = −0.05 (p = 0.27) for human and fly, while the age-regulated fly genes paired with human orthologs show a Pearson correlation r = −0.05 (p = 0.12). (B) Regression slopes of age-regulated genes from human kidney and C. elegans. Open circles denote age-regulated genes in humans and their orthologs in worms. Open triangles denote age-regulated genes in worms and their orthologs in humans. The scatterplot shows the regression slopes from the human kidney and C. elegans aging datasets (Lund et al. 2002). The age-regulated human genes paired with worm orthologs show a Pearson correlation r = 0.05 (p = 0.54). The age-regulated worm genes paired with human orthologs show a Pearson correlation r = −0.01 (p = 0.08). These results show no evidence for overlap in the aging process between different species. (509 KB PDF). [file pbio.0020427.sg004.pdf]
