# Supplementary material for: A Transcriptional Profile of Aging in the Human Kidney
Source: PLoS Biol. 2004 Nov 30;2(12):e427. doi: 10.1371/journal.pbio.0020427 (PMC532391; doi:10.1371/journal.pbio.0020427)
Supplement: Protocol S1 — (40 KB DOC). [file pbio.0020427.sd001.doc]

**Affymetrix HG-U133 Set Gene Chip Protocol**  **List**

**Samples:**

Evening before:

1. Check OD of total RNA and run a final gel.

2. Calculate how much RNA is needed for (8ug) for labeling.

Add that amount to a new tube and speedvac on high until dry.

3. Resuspend RNA in 7.5 ul water and keep at -20C O/N.

4. Heat RNA at 37C for 5 minutes.

5. Vortex and spin briefly.

**Day 1:_______________________**

**cDNA Synthesis**

**First Strand Synthesis**

1. Make master mix of water and T7 Primer:

Master Mix ___x

DEPC-treated water (Ambion) …………3 ___

T7 Primer (Affymetrix)……………………1 ___

Total 4ul

2. Add **4ul** Master Mix to each pcr tube, then add 7 ul RNA, mix by pipetting.

3. Incubate at 70C for 10 minutes, spin, put on ice.

4. Make master mix:

Master Mix ___x

Keep on ICE:

5X **First** Strand Buffer (Invitrogen)……..4 ___

0.1 M DTT (Invitrogen)............................2 ___

10mM dNTP Mix (Invitrogen)…………...1 ___

SSII RT (Invitrogen)……………………...2 ___

Total 9ul

5. Add **9ul** Master Mix to each pcr tube, mix by pipetting.

6. Incubate at 42C for 1.5 hrs, put on ice, centrifuge.

Start:____________ End:___________

**Second Strand Synthesis**

7. Make master mix:

Master Mix ___x

DEPC- treated water (Ambion)…………91 ___

5X Second Strand Buffer (Invitrogen).....30 ___

10mM dNTP mix (Invitrogen)………….....3 ___

10 U/ul DNA ligase (New England Biolabs)…1 ___

10 U/uL **E.Coli DNA Polymerase I**….4 ___

(New England Biolabs)

2 U/uL Rnase H (New England Biolabs)…..0.5 ___

Total 130 ul

8. Add 130 uL to each tube, mix by pipeting, centrifuge, incubate at 16C for 2 hrs.

Start:____________ End:___________

9. Spin down. Add 2 uL [10U] **T4 DNA polymerase** (New England Biolabs), mix by pipeting.

10. Incubate at 16C for 15 minutes.

11. Add 10 uL 0.5M EDTA.

**Cleanup Part I**  (Do in hood)

1. Spin PLG (phase lock gel tube) at full speed for 2 minutes.

2. Add 162 uL of Phenol:Chloroform:isoamyl alcohol (Invitrogen) to new 1.5 mL tube.

(use lower, clear organic layer)

3. Add sample (162 ul) to Phenol/Chloroform tube, vortex.

4. Transfer all liquid to PLG tube, DO NOT VORTEX!

5. Spin at full speed for 2 minutes.

6. To a new 1.5 ml tube, make a master mix:

Master Mix ___x

NH4OAc (Sigma-Aldrich)…………………90 ___

Glycogen (Ambion)………………………4 ___

Total……………………………………94

7. Transfer upper aqueous phase of PLG tube to NH4OAc/Gly tube, finger vortex.

*********be sure to mix everything **well** before adding ethanol!***************

8. Add 600 uL absolute, -20C ethanol

9. Finger vortex and store in -20C overnight.

**Day 2:_______________________**

**Cleanup Part II**

1. Take samples out of freezer and let sit for 15 minutes on ice.

2. Spin 20 minutes at full speed in RT.

3. Add 500 uL 75%, -20C ethanol, vortex, spin at full speed 10 minutes.

4. Pipet out supernatant.

5. Add 500 uL 75%, -20C ethanol, vortex, spin at full speed 10 minutes.

6. Pipet out supernatant and use speed vac to dry pellet.

7. Dissolve WELL in 22 uL DEPC-treated water, spin, incubate at 37C for 5 min and spin again.

**IVT Reaction Setup**

1. Make master mix (Enzo BioArray HighYield RNA Transcript Labeling Kit, Affymetrix):

Master Mix ___x

Solution 1………………………………2 ___

Solution 2………………………………2 ___

Solution 3………………………………2 ___

Solution 4………………………………2 ___

Solution 5………………………………**1** ___

2. Setup and mix by pipetting.

Master mix……………………………..9

cDNA…………………………………...11

Total…………………………………….20

3. Use thermalcycler program set at 37C (~12 hrs): **labeling**

**Day 3:_______________________**

**IVT Reaction Cleanup**

(Qiagen RNeasy Mini Kit)

1. Take samples out of thermalcycler, RT.

2. Label eppendorfs, add 350 **ul Buffer RLT**.

3. Add 80 ul water to IVT sample tube, mix well, add to RLT tube, mix well then pipet from RLT tube to sample tube, mix well again, and then pipet all remaining liquid from sample tube to RLT tube, vortex- PUT RLT away!

4. Add 250 ul 100% ethanol to RLT tubes, mix, then transfer all liquid to RNeasy column.

5. Spin at 8,000 rpm for 1 minute.

6. Dump flow through, add 500 ul **Buffer RPE**, spin at 8,000 rpm for 1 minute.

7. Discard collection tube, put Rneasy column in new 2 ml collection tube.

8. Spin at full speed for 2 minutes.

9. Discard collection tube, put column in new 1.5 ml collection tube.

10. Add 55 ul DEPC-treated water, spin at 10,000 rpm for 2 minutes.

11. Measure OD (use 1x TE).

**Fragmentation and Hybridization**

1. 1 hour prior: Turn heat bath on to 99C.

2. Take OD and calculate uL needed for 15 ug cRNA.

3. Add that amount to a new tube and dry in speedvac.

4. Resuspend in 32 uL water, incubate 5 minutes at 37C.

5. Take chips out of cold room and place on bench unwrapped with window side down.

6. In pcr tube add and mix by pipeting:

5x fragmentation buffer (GeneChip sample cleanup module)…..8

cRNA…………………………………............................32

7. Run thermalcycler program: **frag_94** for 35 minutes.

8. Make master mix (keep on ice):

(5x is max per tube)

Master mix ___x ___x ___x

DEPC-treated water………………………….. 97 ___ ___ ___

2x Hybridization buffer………………………..150 ___ ___ ___

Acetylated BSA (Invitrogen)………………………3 ___ ___ ___

Herring Sperm DNA (Promega)………… ………3 ___ ___ ___

Oligo B2 (Affymetrix)………………………………7 ___ ___ ___

Total……………………………………260 ul

9. Make 300 uL 1X hybridization buffer per chip (from 2X hyb buffer).

10. Label chips on both sides and pierce top septa with pipet tip.

11. Turn on hybridization oven to 45C.

12. Add 260 uL master mix to each eppendorf tube. Add 40 uL of fragmented cRNA to tube with master mix. Vortex.

13. Pierce top of tube with syringe.

14. Put tubes in 99C heat block (filled with water) for 5 minutes.

15. In the meantime make sure the chips are at RT then add 300 uL 1X hyb to each chip and shake the chip slowly to distribute buffer on chip. Leave for about 10 minutes.

16. Spin tubes at full speed for 5 minutes.

17. Remove 1X hyb from chips.

18. Add sample to chip, leaving a small air bubble.

19. Put in hyb over for 16 hours.

Start:____________ End:___________

**Day 4:_______________________**

1. Take chips out of hyb oven.

2. Put pipet tip in top septum to vent and remove hyb cocktail and put in separate labeled eppendorf tube for storage.

3. Add 300 uL Wash A with minimal bubbles.

4. Prime fludics station with Wash A and Wash B.

5. Make master mix for 1^st^ and 3^rd^ stain:

Master mix 2x

DEPC-treated water……………………………………540

2X stain buffer…………………………………………..600

BSA (Invitrogen)……………………………………………48

Phycoerythrin Streptavidin (Molecular Probes) …………..12

6. Vortex and take out 600 uL and put into another tube.

7. Make 2^nd^ stain:

DEPC-treated water……………………………………266

2X stain buffer…………………………………………..300

BSA (Invitrogen)……………………………………………24

Goat IgG (Sigma-Aldrich)…………………………………...6

Biotinylated antibody (Vector Laboratories)………………...3.6

8. Vortex and take all three stains to the fluidics station.

9. Set programs on fluidics station and run the EukGEs2v4 protocol.

10. Load chip and 1^st^ stain and start protocol.

1^st^ stain: 42 minutes

2^nd^ stain: 10 minutes

3^rd^ stain: 27 minutes

11. Check chips for bubbles before scanning. If there are bubbles, empty liquid, wait a few minutes and add more Wash A gently.

12. Place white round stickers on top of vents to prevent leakage into scanner.

13. On new scanner enter expt info and start scan.
